# Coexistence Analysis of 5G C-Band Indoor Deployments and Fixed Satellite Service (FSS) in Malaysia

**DOI:** 10.12688/f1000research.175496.1

**Published:** 2026-06-11

**Authors:** Abdulmajeed Al-Jumaily, Qasim Khalaf, Mohammed Abbas, Yaseein Hussein, Víctor Jiménez, Aduwati Sali, Saba Aljumaili, Dhiya Al-Jumeily

**Affiliations:** 1 Department of Signal Theory and Communications (DTSC), Department of Signal Theory and Communications (DTSC), Charles III University of Madrid, UC3M, Leganes, Madrid, Leganes, Spain; 2 Construction and Projects Department, Construction and Projects Department, University of Fallujah, Al-Fallujah 31002, Iraq, Fallujah, Anbar, 31002, Iraq; 3 Department of Computer Engineering Techniques, Department of Computer Engineering Techniques, College of Technical Engineering, University of Al Maarif, Al Anbar,31001, Iraq, Ramadi, Anbar, 31001, Iraq; 4 Department of Information Systems and Computer Science, Department of Information Systems and Computer Science, Ahmed Bin Mohammed Military College, Doha, Qatar, Doha, Qatar, Iraq; 5 WiPNET Research Centre, Dept of Computer & Communication System Engineering, WiPNET Research Centre, Dept of Computer & Communication System Engineering, Faculty of Engineering, Universiti Putra Malaysia, 43400 Serdang, Selangor, Malaysia., Serdang, Selangor, 43400, Malaysia; 6 Genetics and Molecular Biology eSystems Engineering Society, Genetics and Molecular Biology eSystems Engineering Society Malaga, Spain, Malaga, Spain; 7 Department School of Computer Science & Mathematics, Department School of Computer Science & Mathematics, Liverpool John Moores University, UK., Liverpool, UK

**Keywords:** 5G NR (New Radio) frequencies, Fixed Satellite Service (FSS), C-Band, Indoors measurements

## Abstract

Several satellite services, including the Fixed Satellite Service (FSS), rely on the C-Band (3. 4–4. 2 GHz), which provides wide coverage and high availability despite weather effects. As a mid-band spectrum ideal for 5G, its allocation to cellular networks raises interference concerns, potentially disrupting satellite services and causing a significant economic impact. This paper presents a 5G propagation model and an interference investigation with 5G cellular networks operating below the 6 GHz band in the optimization of exclusion zones for 5G and FSS coexistence scenarios. To fine-tune the exclusion zone and identify better conditions between 5G and FSS. We provide an extensive 5G-cellular downlink analysis that considers potential Low-Noise Block (LNB) saturation at the FSS Earth station receiver. Moreover, we put new 5G network-based cellular networks into practice. Prior to the power signal transmission ceasing the network, high-antenna receivers were employed in both indoor and outdoor settings at various locations using the 5G base station transmitter to receive signal input from inside the proposed location. Additionally, a spectrum analyzer was used to examine the power signal received in contrast to the data signal acquired for the proposed projects. According to the findings of this study, spectrum regulators and other pertinent stakeholders will be impacted by the possible deployment of 5G cellular networks in C-band operating FSS Earth station receivers. The study also suggests and assesses strategies that might be used to encourage the coexistence of both systems, such as turning off important emitters or reducing their transmission capacity.

## 1. Introduction

**Figure 1.**
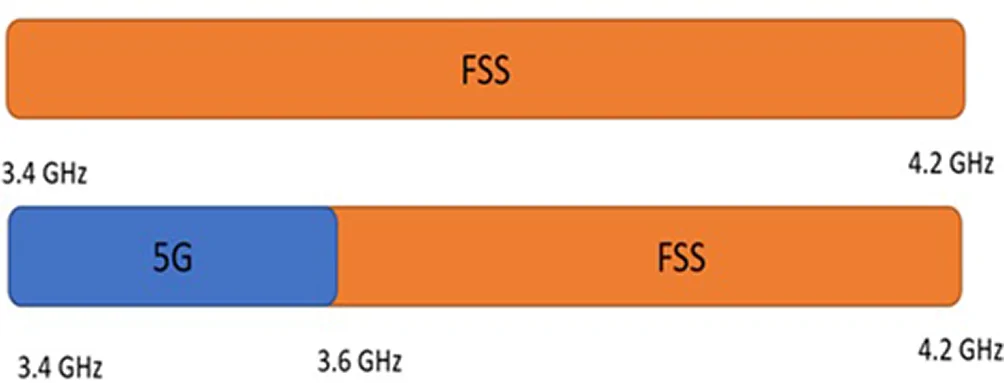
Proposed frequency arrangements for C-Band in Malaysia.

5G refers to a fifth-generation mobile network. Furthermore, the following standards were adopted and published approximately every nine years since the establishment of the first mobile network in 1982: GSM, the second generation of cellular networks, was first released in 1992, and rival 3G specifications were introduced in 2001. ^1, 2^ Service providers used the 4G LTE cellular standards in 2010. To create new mobile devices, technology companies and mobile operators worldwide are now using 5G technology. These 5G implementations are followed by LTE transformation technologies such as LTE Advanced and LTE Advanced Pro, which allow network operators to deliver faster speeds on mobile devices. ^3^ Ericsson published its 2017 “5G Readiness Survey.” The operators sped up preparations for the new technology, and 78 percent of the respondents were conducting trials. In addition, 28 percent expect the fifth generation to be deployed in 2018. App interactions are evolving rapidly. Cisco VNI Mobile data show that mobile data traffic is expected to reach 11 exabytes per month in 2017, double that in 2015, reaching 49 exabytes by 2021. As of July 25, 2019, 5G was introduced into commercial use on 27 worldwide networks wished to deploy more than 150,000 base stations of 5G. ^4^ The mobile communication industry is undergoing massive transformation every day. ^5^ New generations and standards have been established to provide registered subscribers with voice, data, and multimedia services at an increased speed. The introduction of new wireless technologies and the rising demand of consumers have enabled the mobile industry to transition from the second to the fourth generation. A decision was taken at the 2015 World Radio Communication Conference to designate 3.3 GHz – 4.2 GHz (3.5 GHz C band) to future mobile broadband, 5G beamforming, and 5G multi-antenna structure. This encourages service providers to use this band for future 5G systems to ensure easy deployment. ^1^ For this paper was focus on indoor frequency 3.5GHz band, especially from 3.4 GHz to 3.6 GHz, as shown in Figure 1. This mid-band spectrum (<6 GHz) is taking shape as the core band for 5G worldwide because of the technical feature that affords an optimal balance of large capacity (amount of supported traffic) and coverage (distance of travelled signal). ^6, 7^

There is a possibility for adverse frequency interference with the planned allocation of 5G networks and FSS systems owing to the technological complexity of the FSS service within the 3.5 GHz range. ^8^ This is reflected by permitting unnecessary signals from 5G base stations and mobile terminals to interfere with FSS earth stations receiving signals in the 3.5 GHz band. ^9– 11^ Technical studies were performed to evaluate the feasibility of the deployment of 5G within the range of the FSS system. This scientific study aims to determine the appropriate guard band, emission power limits, and separation distance. It is necessary to reduce interference and allow the coexistence of the two systems. Such approaches are recommended for testing by theoretical research and field trials based on global best and current practices, as these strategies can be used as a solution to interference management for both local and cross-border scenarios.

## 2. Experimental setup

**Figure 2.**
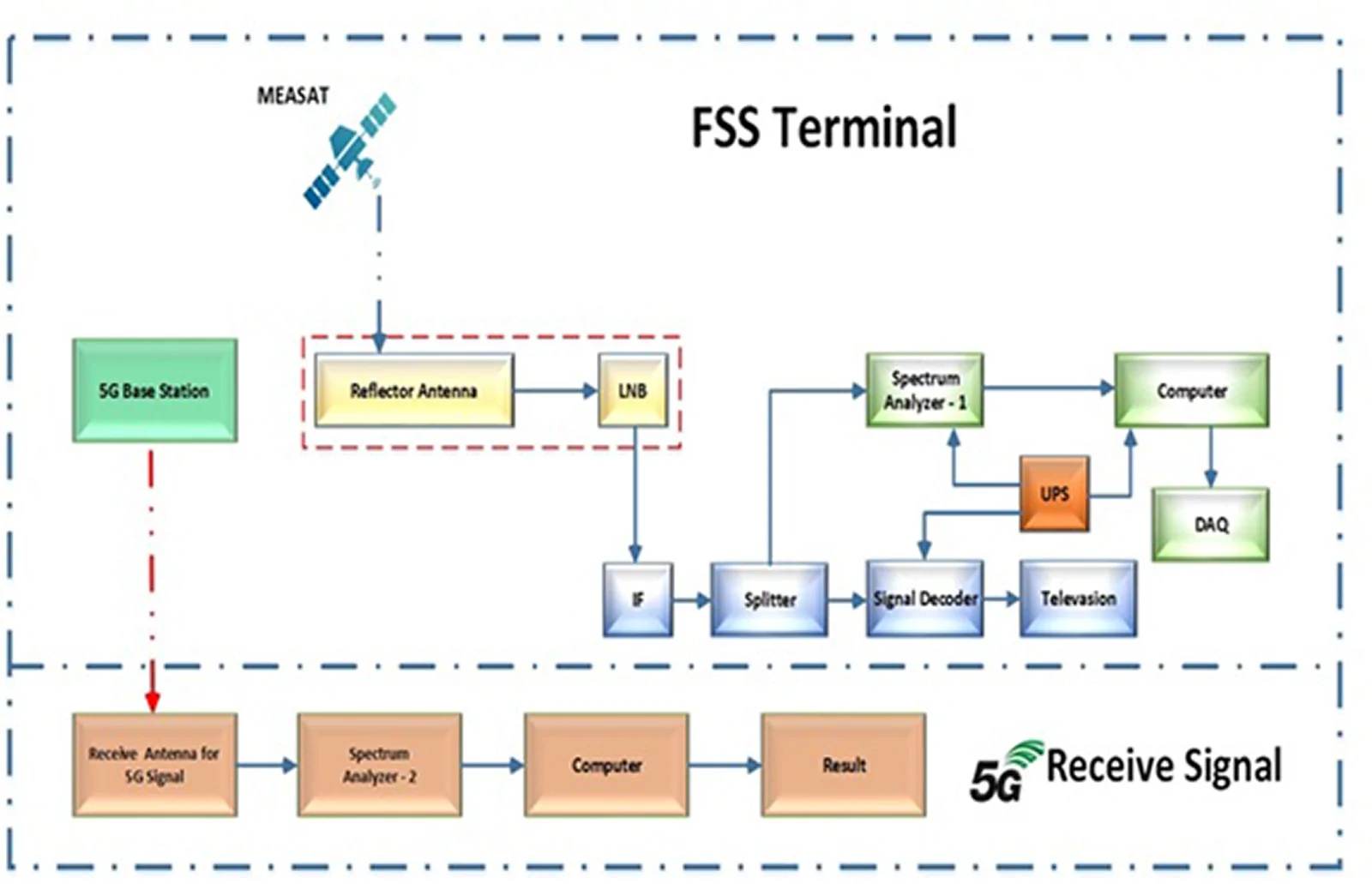
Experimental setup scheme.

The measurement component content of essential high-quality devices provides the best results without any errors. A new beamforming antenna for the 3.5 GHz band (3.3 to 3.8 GHz) was released by radio frequency systems (RFS). The ability to support 3.5 GHz applications is critical as operators worldwide are trying to meet the growing demand for LTE, deploy new small cells, and plan for 5G rollout. The 3.5 GHz band is the preferred sub-6 GHz spectrum for 5G networks worldwide rollout. The base station designed by ZTE is shown in Figure 2. It has the following description of the range of power transamination between (35–10) dBm (3–0.25) watts and the propagation of the signal is approximately 75 m. The purpose is to ensure that the base station operates indoors. Fifth-generation (5G) technology relies primarily on high-capacity transmission that utilizes short-range high-frequency radio waves. ^12^ This makes the 3.5 GHz band ideal for delivering high data rates with stable indoor coverage. In addition, the beamforming capability enhances the signal strength and minimizes interference, thereby ensuring a more reliable communication performance.

### 2.1 Location of measurement for received signal

**Figure 3.**
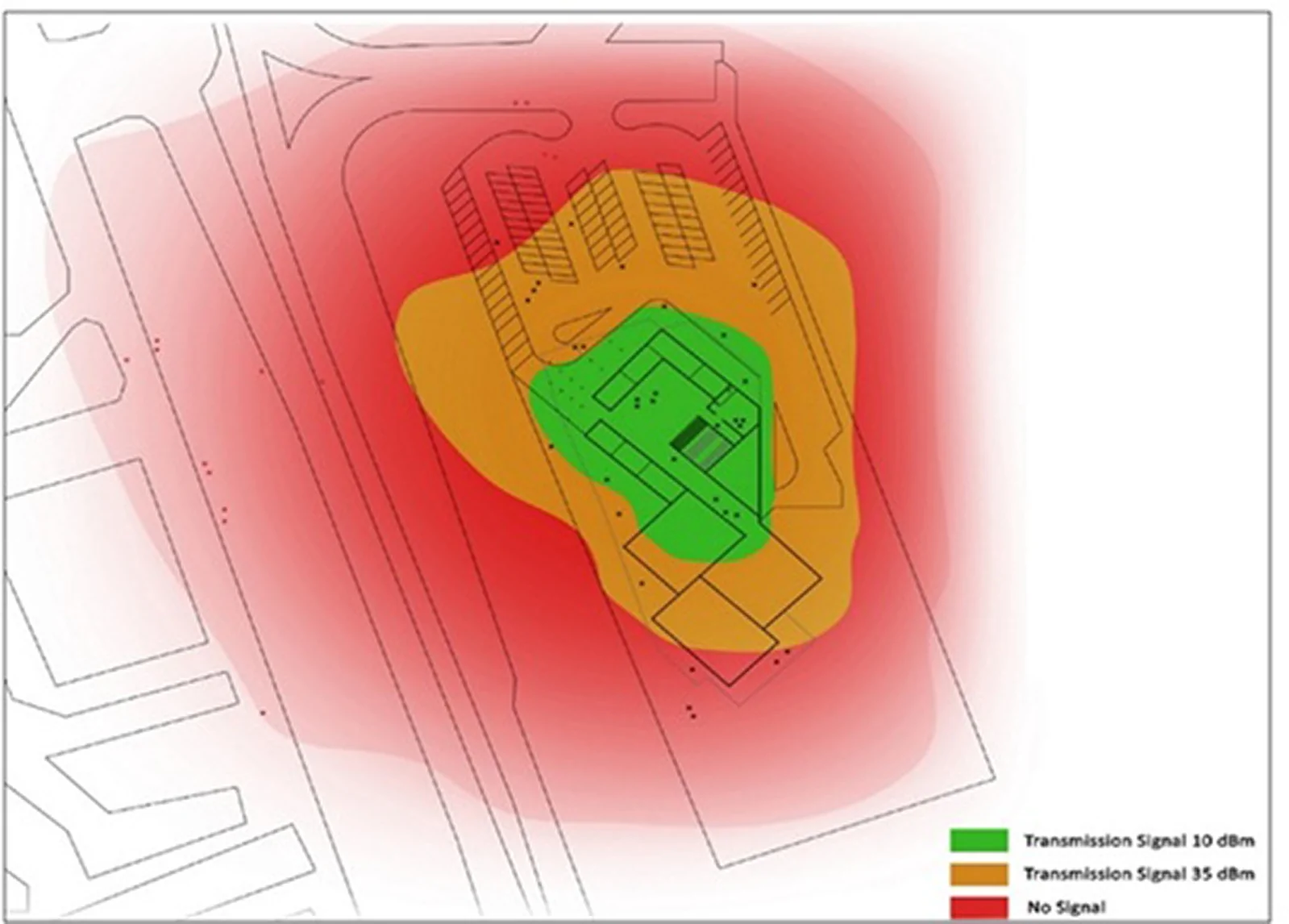
Map 1 for RekaScape building.

**Figure 4.**
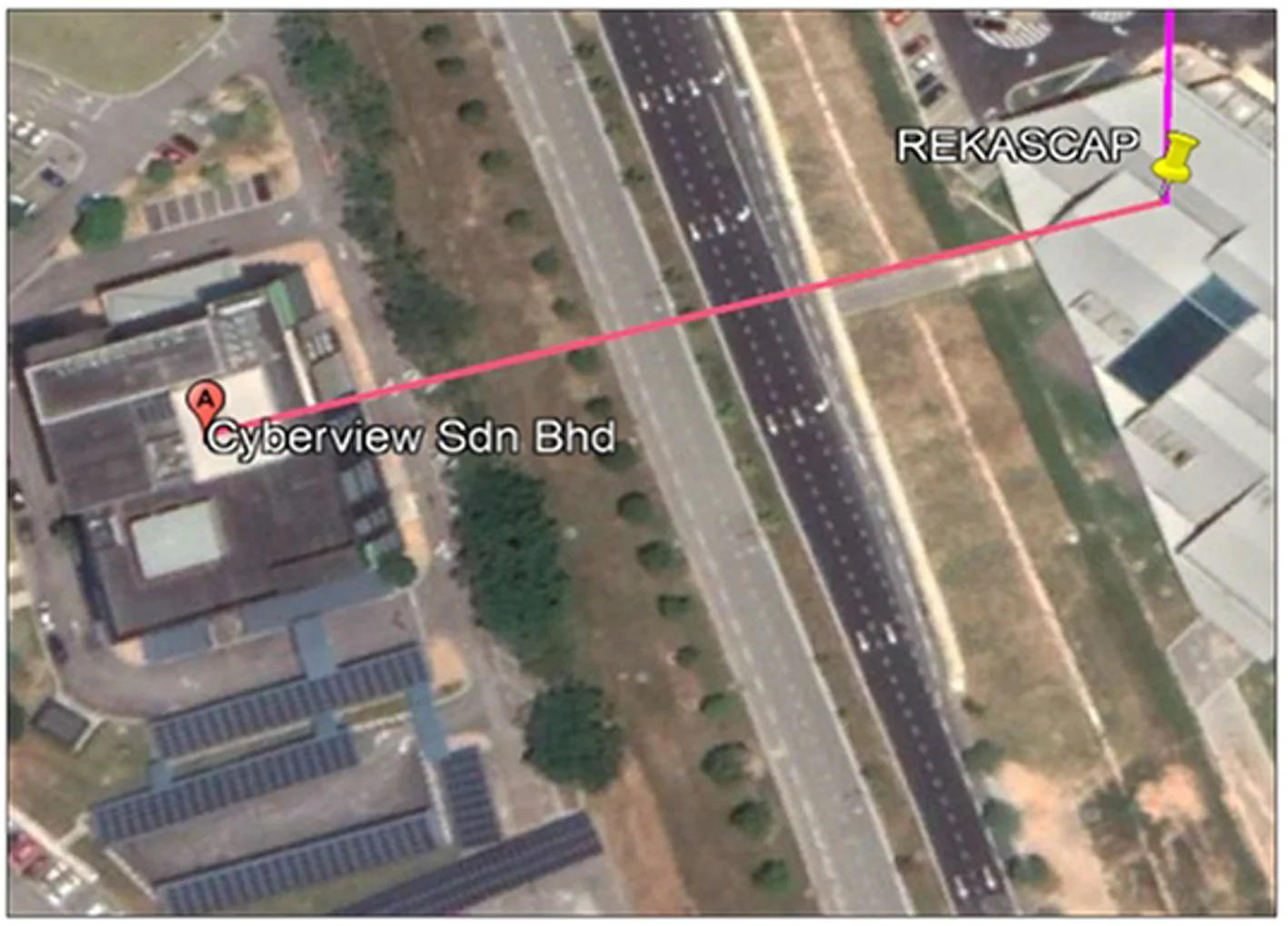
The distance of received signal (130 m), from RekaScape to Cyberview.

**Figure 5.**
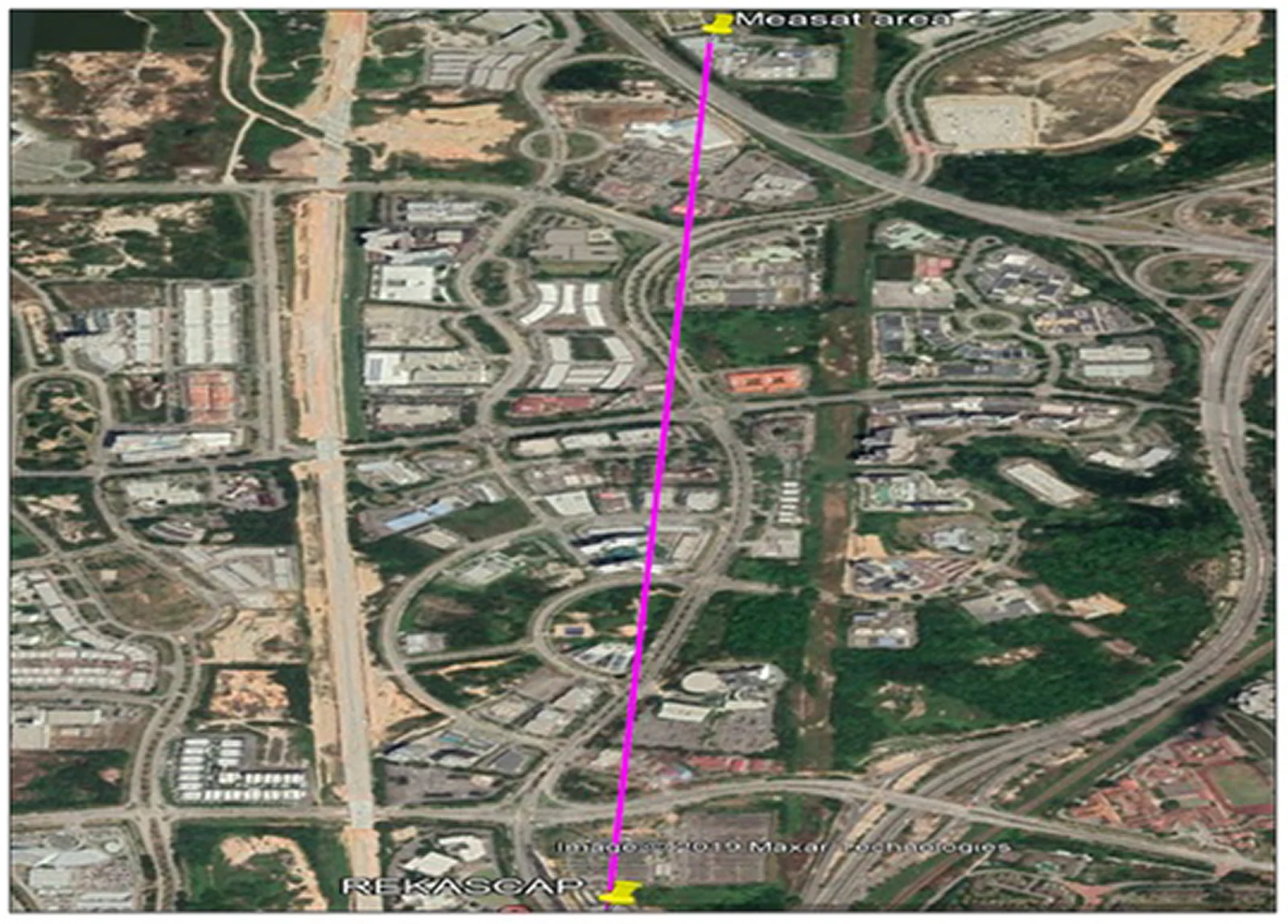
The distance of received signal (3 km), from RekaScape to MEASAT.

This section describes indoor measurement scenarios. As shown in Figure 3, the location of the indoor scenario was in the RekaScape building and parking. There are also two paths: result line-of-sight propagation (LOS) and non-line-of-sight propagation (NLOS). On the other hand, the interference effect with various distances in parking and crossing the road with a distance (130) meters nearest the building of Cyberview and the other distance was nearest to the MEASAT station with a distance of 3 km. The FSS signals used in this study are shown in Figures 4 and 5.

### 2.2 Receiver antenna parameters

The description is shown in Table 1, where the linear polarized Periodic Broadband Antenna Logarithmic (aluminum tubing) for receiving and transmitting applications. ^13, 14^

**Table 1.** The specification of receiver antenna.

Specifications	Typical
The nominal spectrum of frequency	800 MHz-5 GHz
The frequency spectrum can use	650 MHz-8 GHz
Gain isotropic	4–7 dBi
The factor of the antenna	23–38 dB/m
Typical SWR standing wave ratio	<1.5
Front to Back Ratio	20 dB
Cross-polarization	>20 dB (800 MHz-2 GHz)
Input max power	300 W @ 1 GHz \ 150 W @ 5 GHz

Moreover, the signal intensity variation was close to the location. In addition, the indoor radio transmission system varies significantly from the conventional cellular radio channel in terms of defended lengths. Relevant features, such as the building structure and positions of the antennas, affect propagation inside buildings. The location of the 5G base station inside the ZTE organization hall is 2 m higher from the ground, polarization vertical, and a transmit power of 35 dBm, as shown in Figure 7, and the yellow area covers the position of the points receiving the signal from the 5G station with a range distance between 1 m to 40 m.

**Figure 6.**
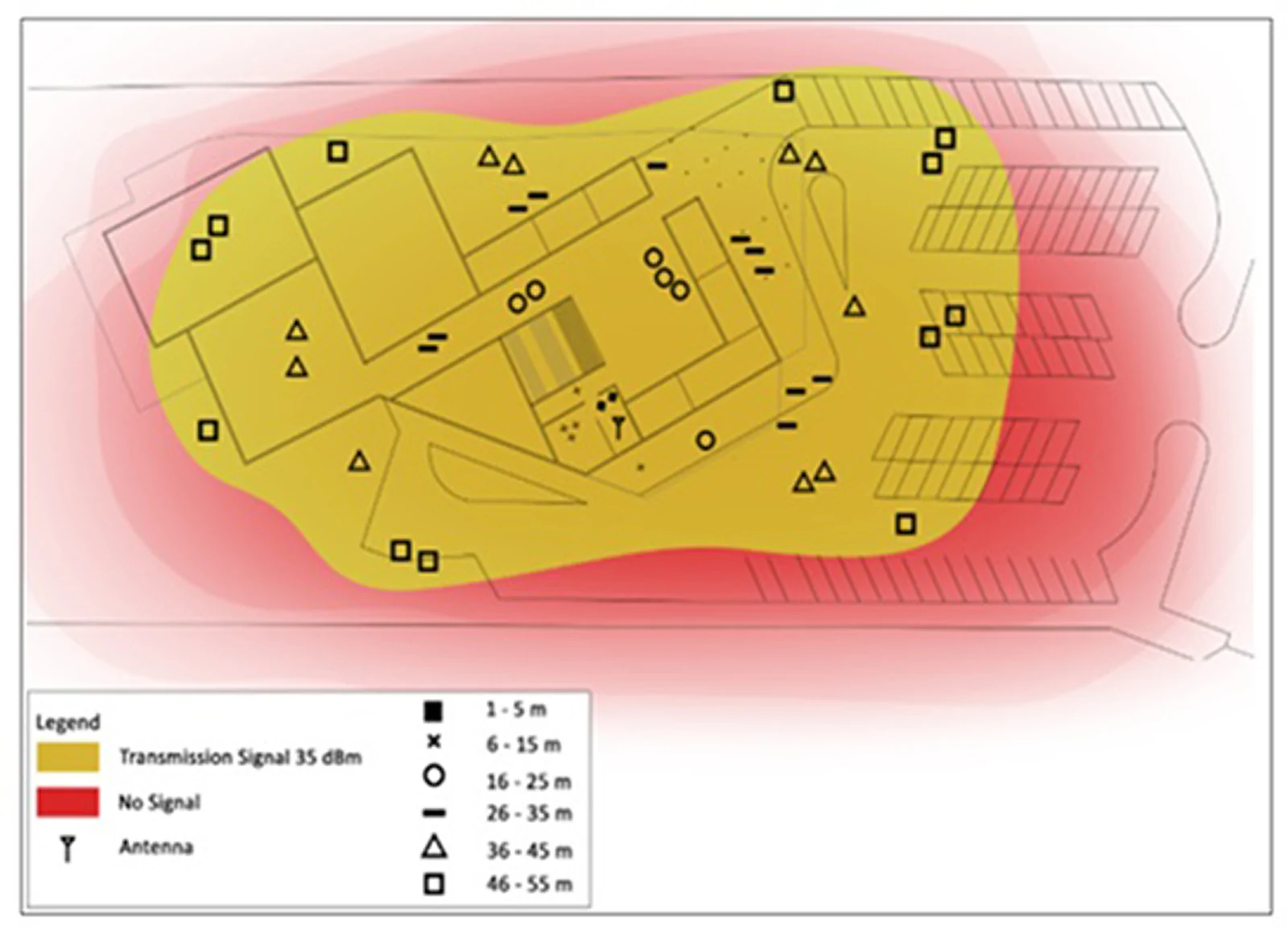
Map 2 5G points locations in RekaScape building Cyberview.

Otherwise, as shown in Figure 6, the gap between the 5G base station and receiver antenna sites is approximately 5 m, with a special symbol (■). The display block settings were as follows: RBW =1 MHz, VBW = 1 MHz, and SWT = 100 ms. The resolution bandwidth (RBW) specified as the frequency range of the final filter that extends to the input signal. Relatively small RBWs provide more natural frequency resolution and the ability to separate signals with closer frequencies. ^15^ Another aspect influencing a spectrum analyzer is that trace efficiency is the video bandwidth (VBW). This noise makes it difficult to detect tiny signals when VBW is high. As we lower the VBW, the small signal is even more evident. The sweep time (SWT) is the amount of time it takes from the start to the stop frequency from sweeping the detector. This noise makes it difficult to detect tiny signals when VBW is high. As we lower the VBW, the small signal is even more evident. The sweep time (SWT) is the amount of time it takes from the start to the stop frequency from sweeping the detector. The (SWT) value can be determined from the following equation: $\mathrm{SWT}=\frac{K_{T}+\text{SPAN}}{{\mathrm{RWB}}^{2}}$ ; the time required affects the RBW and VBW ratio. The VBW is typically set to a value greater than or equal to the RBW when we do not rely on noise, whereas the principal effect of the VBW and RBW detectors is to smooth the tracing and noise, ^16^ Moreover, innovations in RF receiver front-end design, encompassing rectifier technologies and energy harvesting architectures for simultaneous wireless information and power transfer (SWIPT) systems, have illustrated that shared-spectrum environments necessitate meticulous attention to receiver sensitivity, multi-band operation, and power conversion efficiency to guarantee dependable coexistence among diverse wireless services. ^17^

## 3. Experimental results

In comparison, the frequency ranges start at 3 GHz and end at 4 GHz with a duration width of 1GHz at the middle scale of 3.5 GHz.

### 3.1 Measurement result

**Figure 7.**
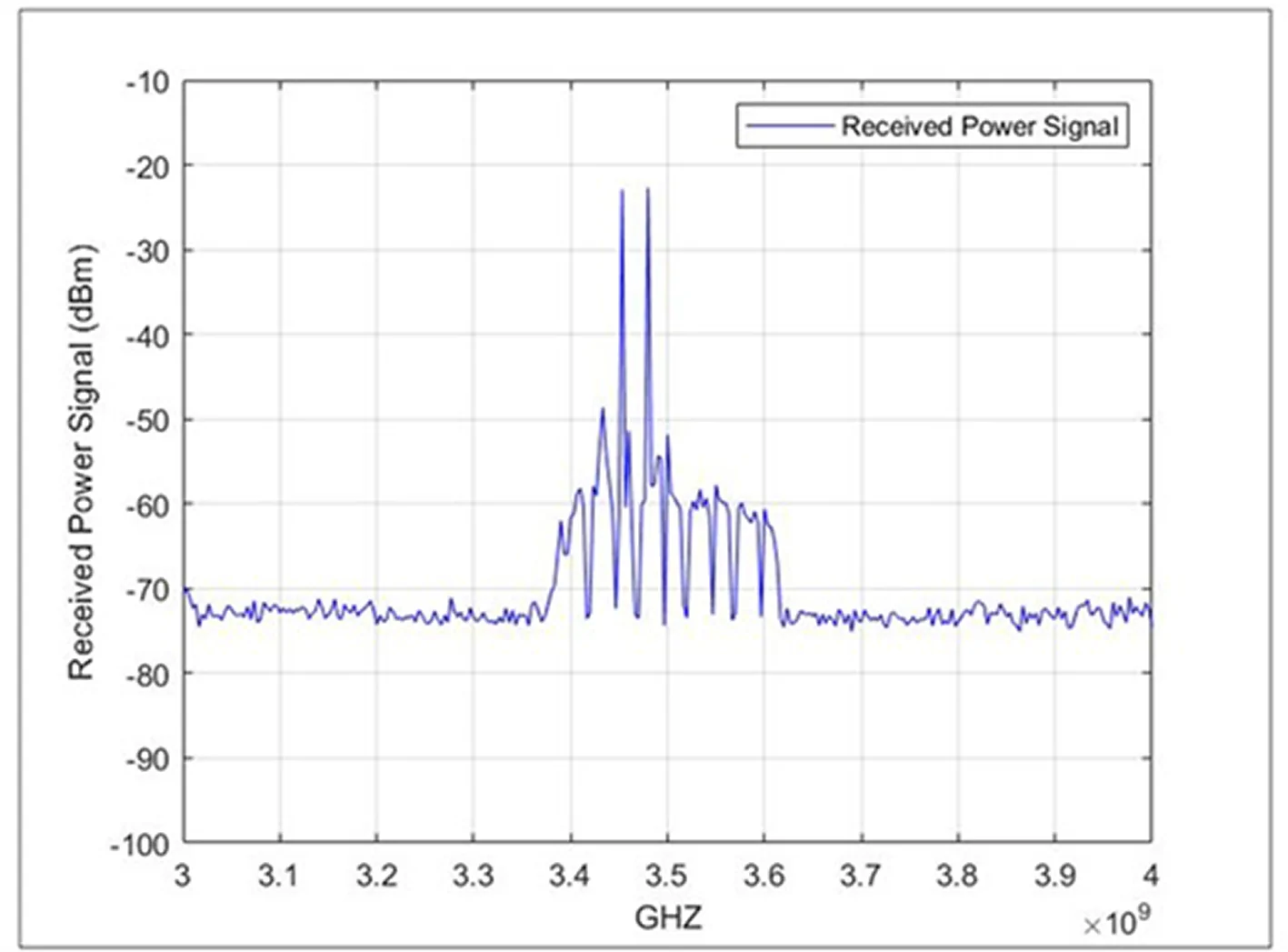
Experiment result with distance 1 to 5 m.

The first results, as shown in Figure 7, indicate that the particular range in the received power has the highest signal, with frequency of 3.49 GHz, −54.34 dBm, in the same lap as the 5G transmission station, where the gap between the two sites is approximately 1 to 5 meters.

**Figure 8.**
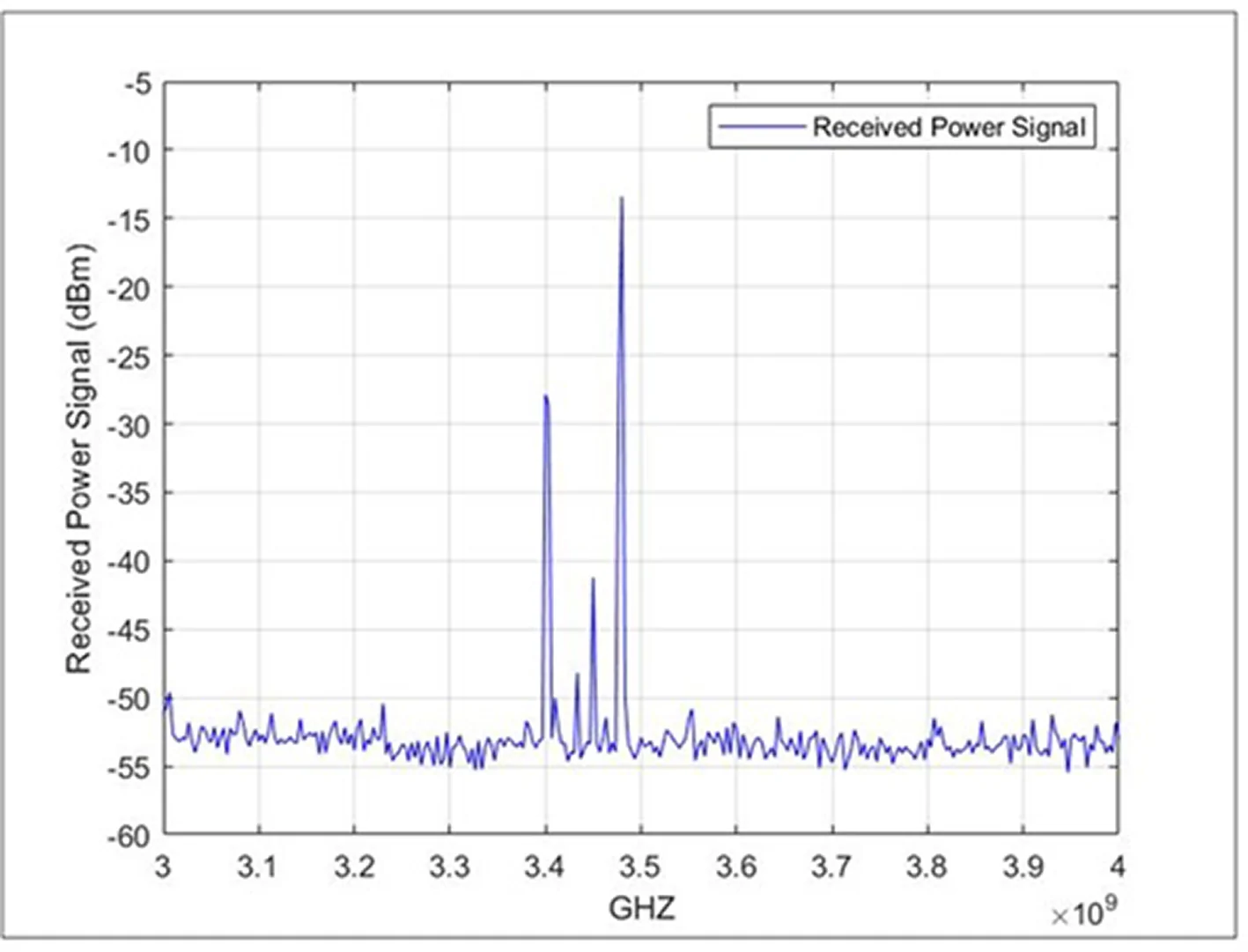
Experiment result with distance 10 m.

Figure 8, the signal is in the smooth mode from 3 GHz to 4 GHz with −50 dBm power level. The first peak received at the frequency 3.4 GHz with power − 30 dBm, and at the 3.48 the system received the highest peak in the −13.41 dBm from the received power signal with distance 10 m from the antenna of 5G station.

**Figure 9.**
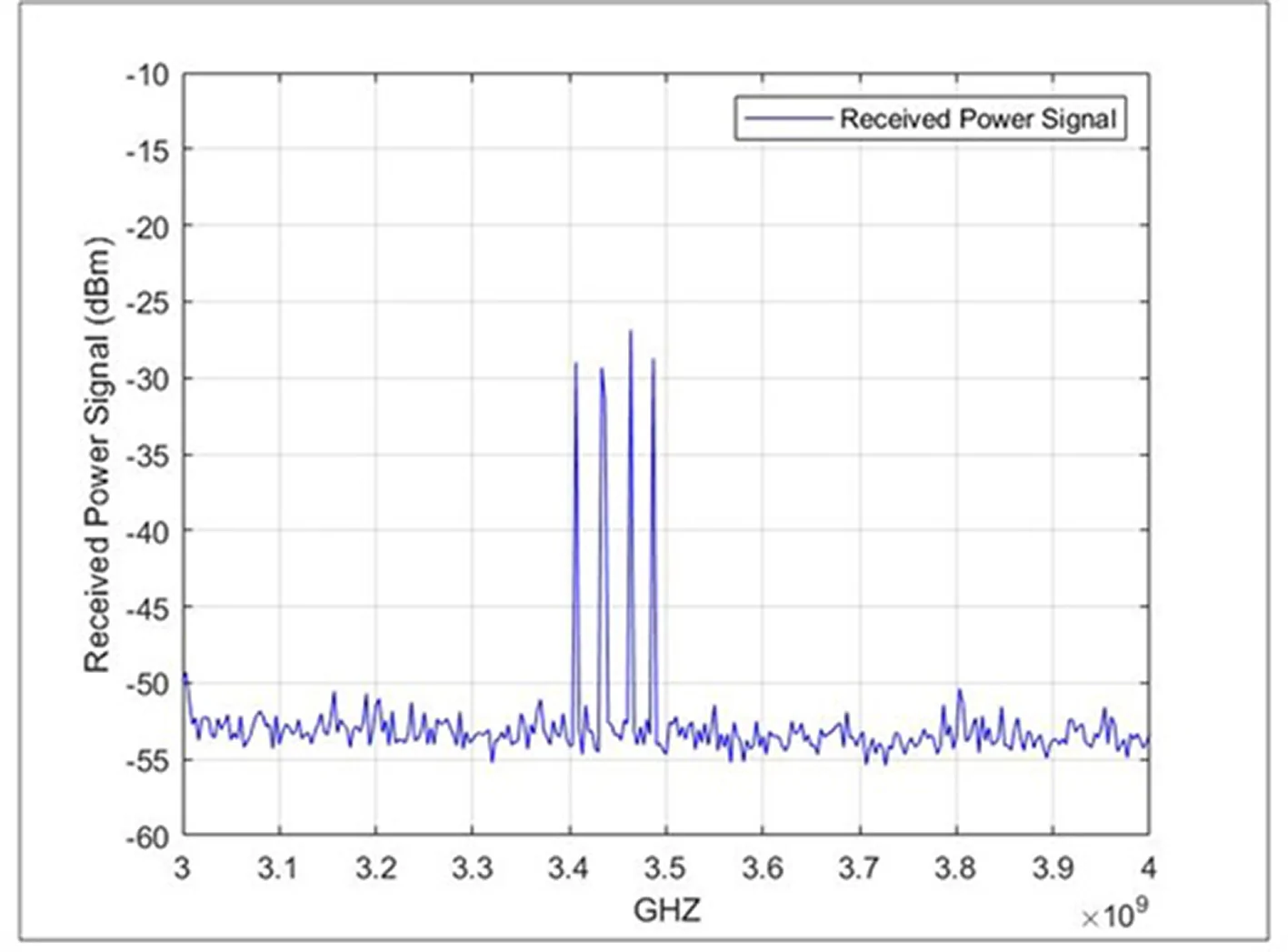
Experiment result with distance 20 m.

Figure 9, demonstrates that the first three puls received starts from 3.4 GHz to 3.5 GHz. The highest peak of the received power signal from the 5G station antenna at −28.41 dBm, with a distance of 20 m.

**Figure 10.**
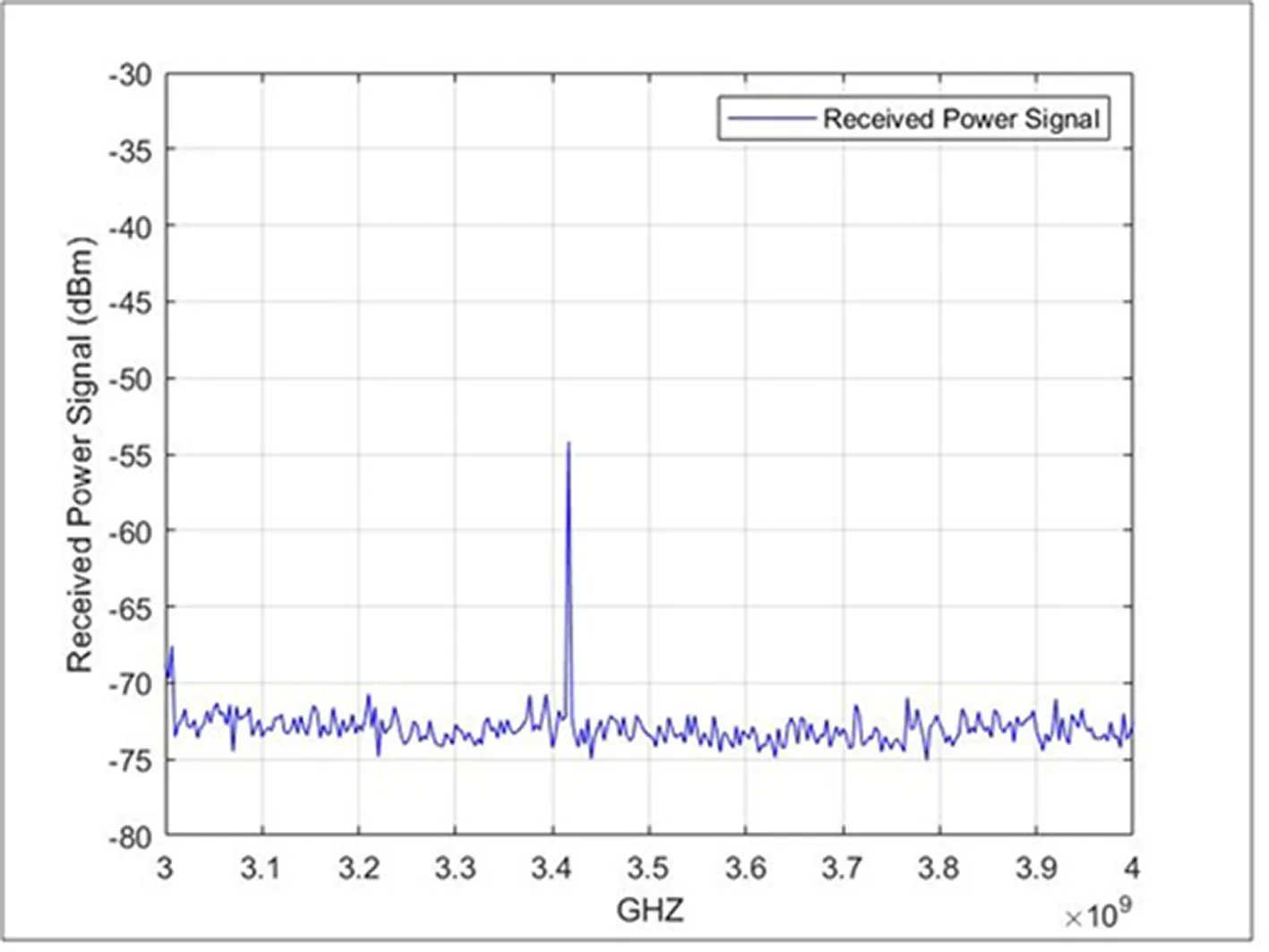
Experiment result with distance 30 m.

Figure 10, at a frequency of 3.42 GHz, indicates the maximum hold that will be obtained from the 5G station antenna at −54.61 dBm at the 30 m distance.

**Figure 11.**
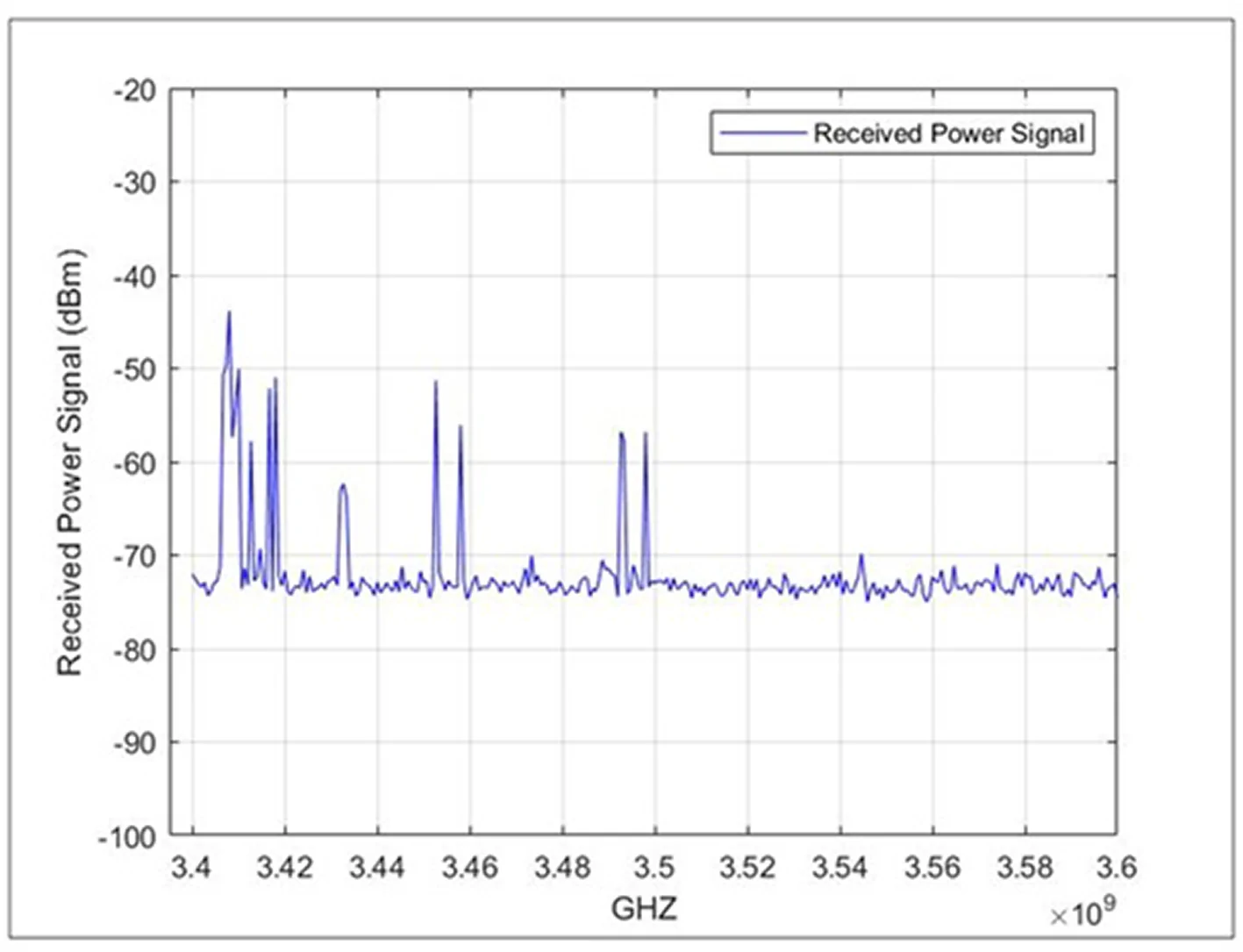
Experiment result with distance 35 m.

Figure 11, at a frequency of 3.41 GHz, shows the highest control received from the 5G station antenna at −52.34 dBm over a distance of 35 m.

**Figure 12.**
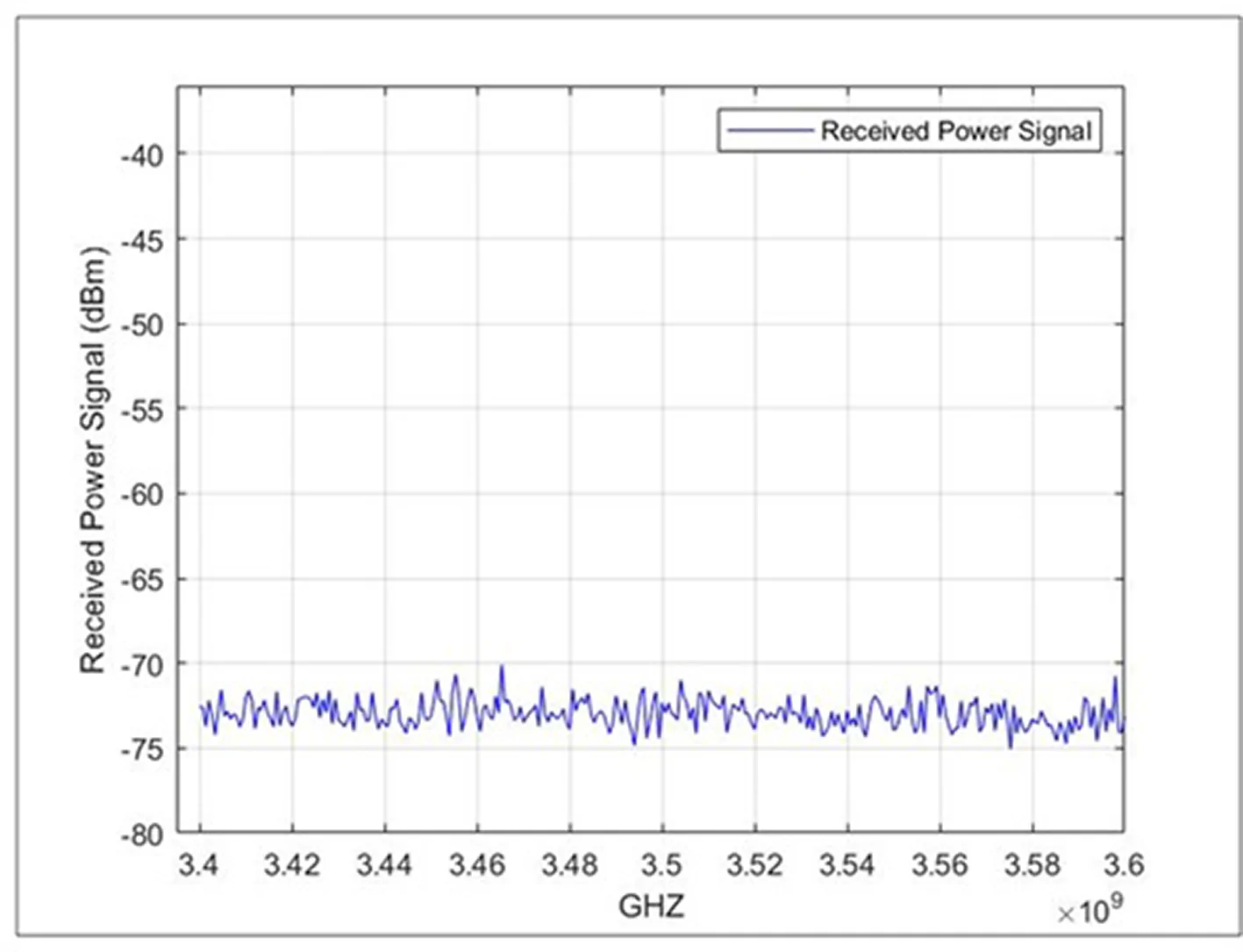
Experiment result with distance 40 m.

Figure 12 indicates that the signal is prevented by the cement wall between Tx and Rx. This point was collected from the 5G antenna at the highest distance. The first peak appeared within the initial range of 3,456 GHz at a power level of −74.02 dBm.

### 3.2 The distance model between Tx and Rx antenna

**Figure 13.**
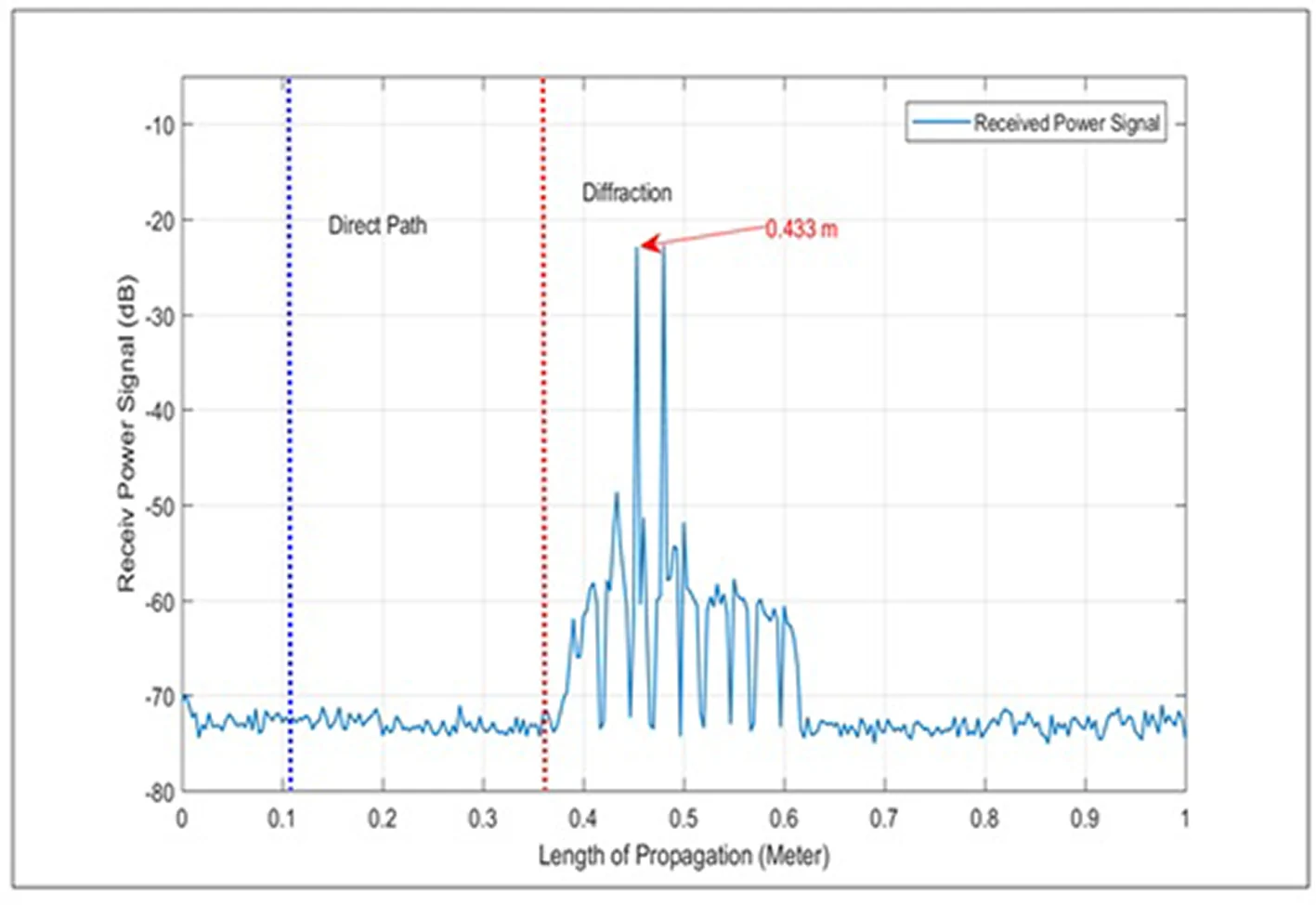
Distance of 1 m (Tx to Rx) Antenna.

This section explains the comparison between the received power signals and propagation length (m). Figure 13 shows the maximum receiving power transmitted by the higher 5G antenna transmission approximately at 0.43 m with approximately 35 dBm.

**Figure 14.**
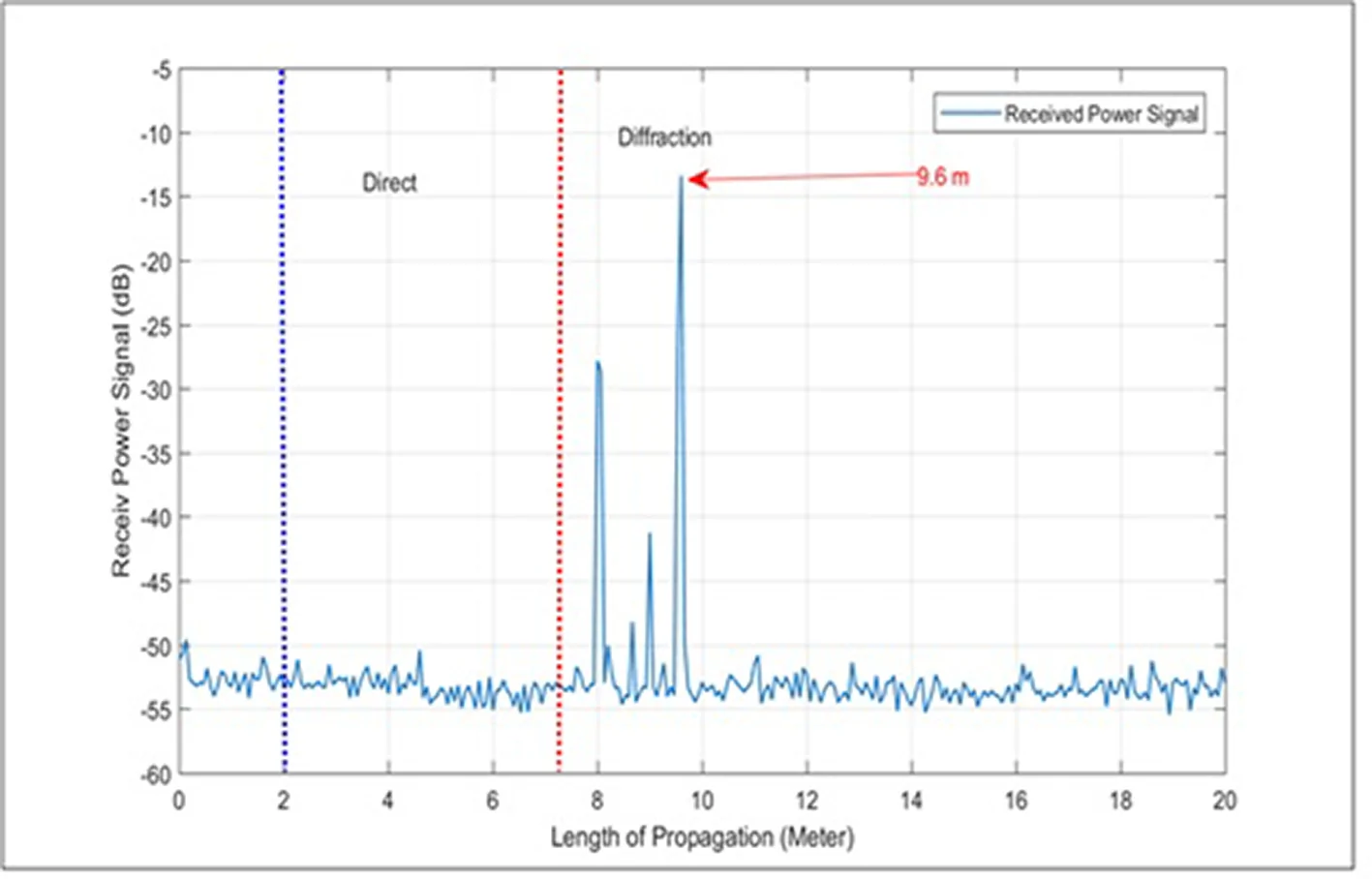
Distance of 10 m (Tx to Rx) Antenna.

Figure 14 indicates the highest signal at 10 m with −13 dBm, while at the 20 m the power received was less than approximately −30 dBm.

**Figure 15.**
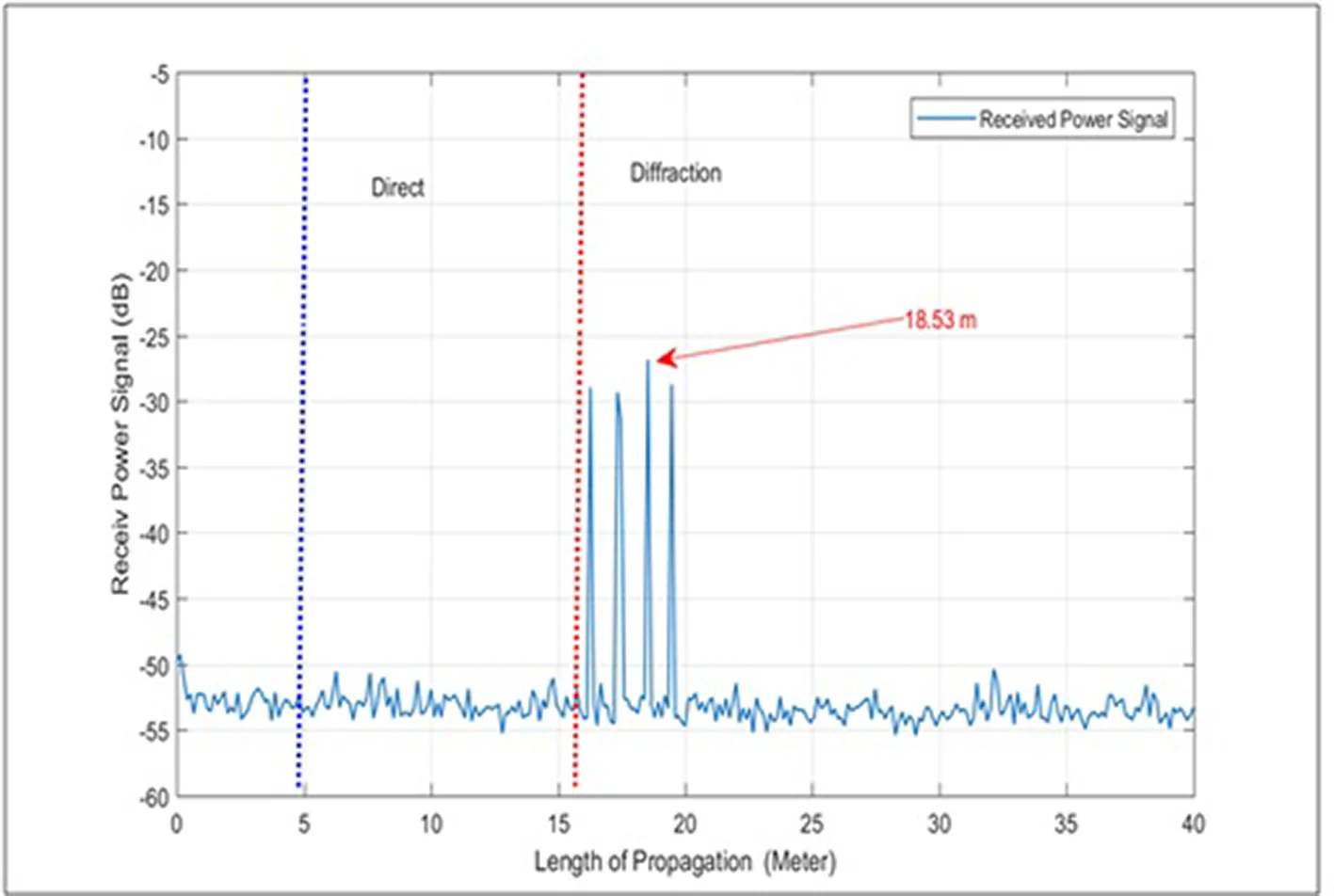
Distance of 20 m (Tx to Rx) Antenna.

Figure 15 shows the received signals at different distances and antenna transmissions, where the transmission power approximately is −32 dBm to −54 dBm, at 20 m.

**Figure 16.**
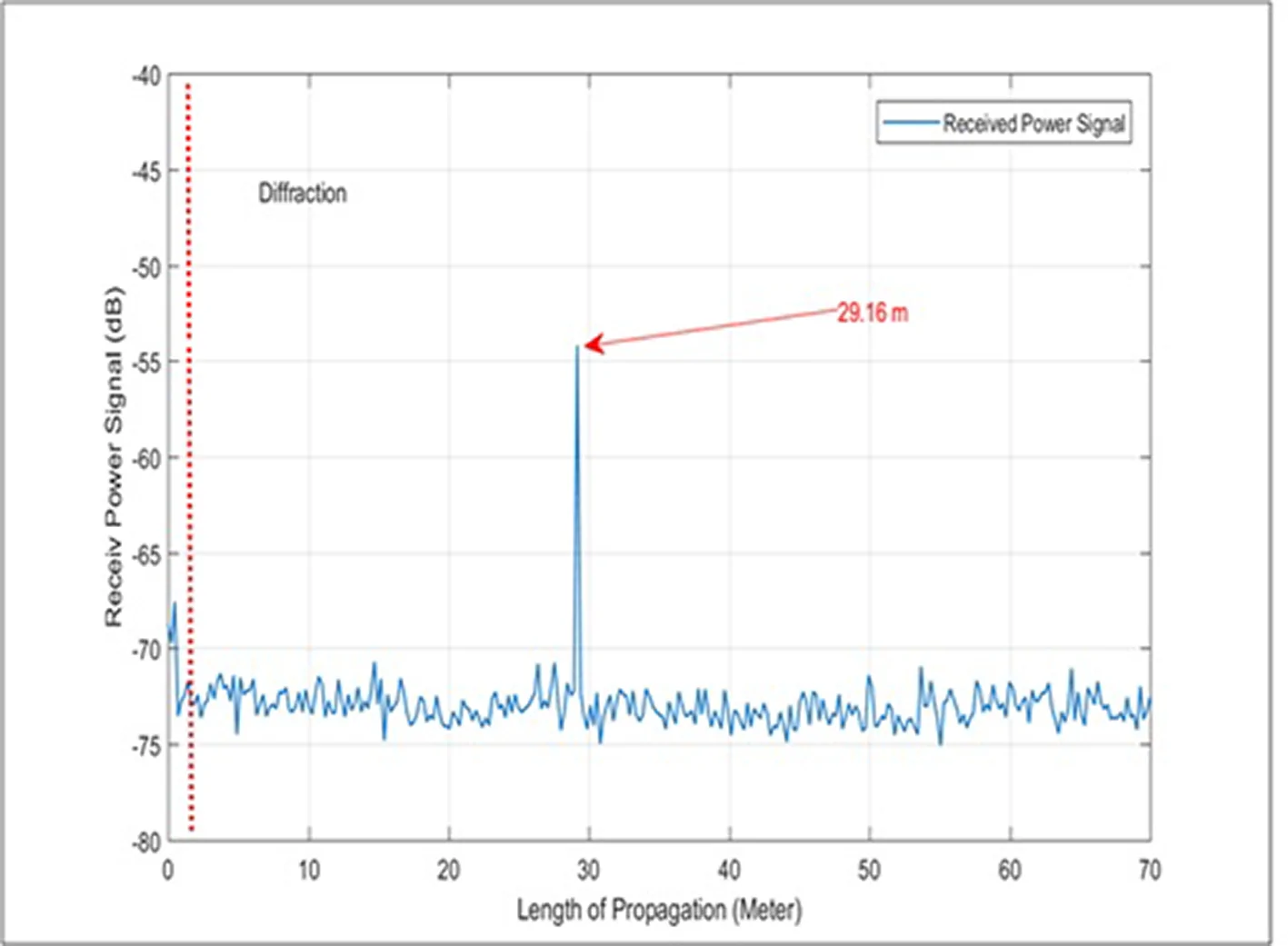
Distance of 30 m (Tx to Rx) Antenna.

Figure 16 shows that the receiving signal is low at approximately − −55 dBm to −74 dBm at 30 m.

**Figure 17.**
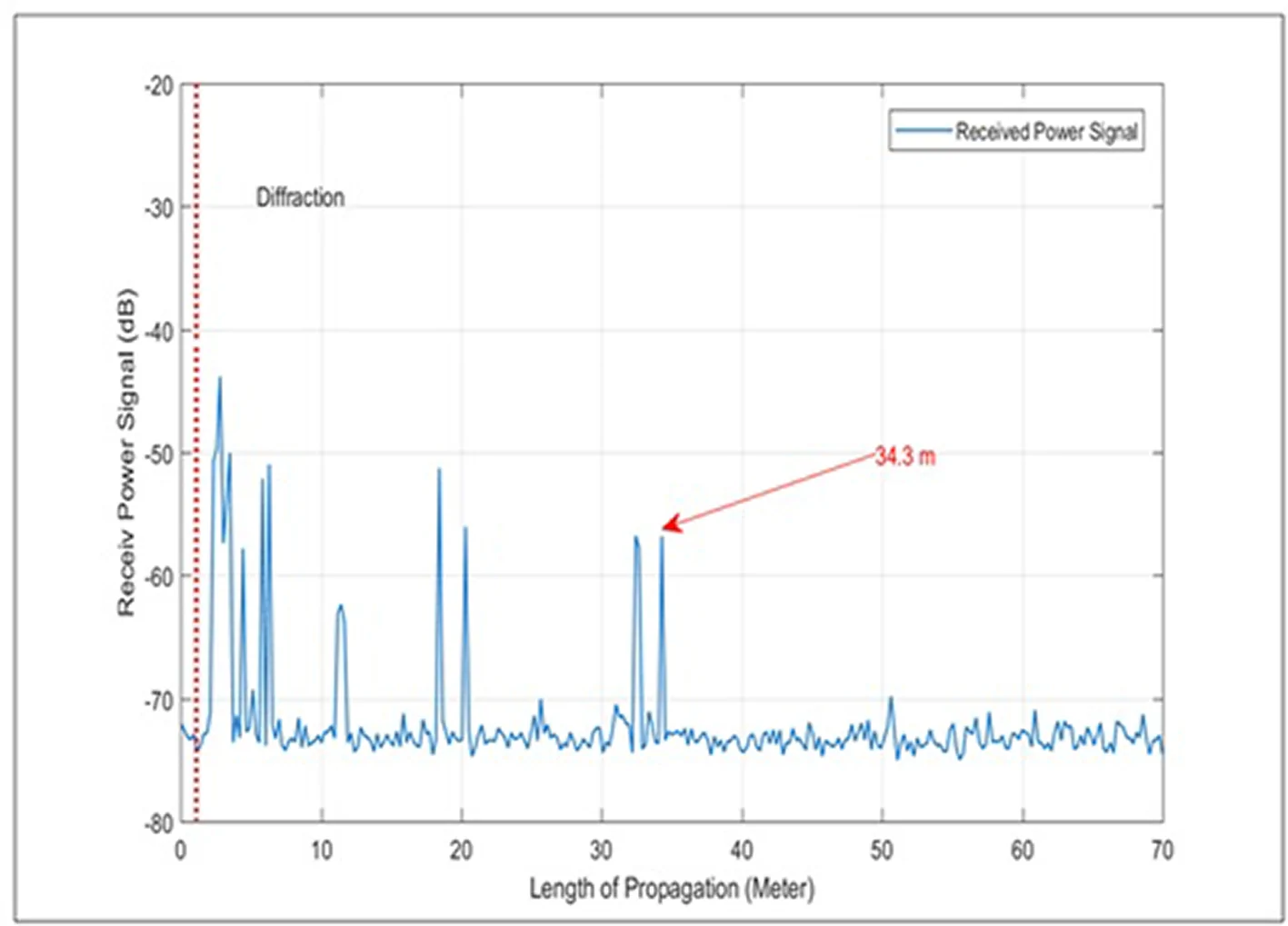
Distance of 35 m (Tx to Rx) Antenna.

Figure 17 shows that the received power signal transmission by the service antenna signal is exceeded. Moreover, at 35 m the power received was approximately −63 dBm to −72.19 dBm.

The optimal exclusion zone between the 5G indoor model and FSS Inter-cell interference proved to be the most significant restricting factor at high altitudes; therefore, many industries and researchers are seeking to use less congested radio components in the total spectrum. The 3.4–3.8 GHz (C-band) is one of the world’s most significant pioneer frequencies for the early launch of 5G networks. However, this band is already being used for fixed satellite services (FSS) in Malaysia and is not significantly affected by rain. The coexistence of mobile broadband networks should be investigated to better define the business conditions in specific environments. This section examines and demonstrates the numerical and measurement results for 5G indoor locations. It is notable from the last received data that there is no interference between the 5G signals and FSS spectrum. This study considers the following parameters, as explained in Table 2.

**Table 2.** Typical parameters values.

Parameters	Values
Power transmission for 5G station	10 dBm, 35 dBm
Location of the 5G station	Inside the RekaScape building and parking
Frequency bandwidths	3.4–3.6 GHz
The max distance the signal receives	35 m
RekaScape closing site using FSS (MEASAT)	3 Kilometers
MESAT frequency bandwidths	>=3.700 MHz

It is notable from Table 2 that the best optimization exclusion zone for the indoor scenario, the distance between the 5G indoor station and FSS station should be approximately 100 m and the forbidden band from 50 to 100 MHz should be between the usage frequency.

## 4. Conclusions

This study achieved all the stated objectives. The results obtained from the study and observations are described and analyzed in this study. Initially, the performance evaluation of 5G transmission with a power equal to 35 dBm resulted in higher data receiving wide-spread power recording. The power output of the 5G indoor base station decreased to 10 dBm owing to a reduction in the scatter size. The range of power received started from −3.53 to −57.88 dBm when the power transmission was high. The nearest center using the FSS transmitted and received signals was the MEASAT. The lowest frequency used by MEASAT is 3.7 GHz, and the distance from the indoor antenna in Rekascape is approximately 3 km. Finally, there is no interference between the 5G indoor signal and FSS signals in the scenario measurements.

## Data Availability

The data supporting the findings of this study are openly available in Figshare at: [DOI: 10.6084/m9.figshare.31872805]. ^18^ The dataset includes radio-frequency measurement data collected during on-site indoor measurement campaigns, along with the processed data used for analysis and figure generation. Additional metadata describing the measurement setup, environmental conditions (e.g., building structures, terrain characteristics, and weather conditions), and experimental parameters are also provided to support interpretation and reuse. The dataset is made available under a CC0 Public Domain Dedication/CC-BY 4.0 license, permitting unrestricted use, distribution, and reproduction in any medium, provided appropriate credit is given.
