# Supplementary material for: Coexistence Analysis of 5G C-Band Indoor Deployments and Fixed Satellite Service (FSS) in Malaysia
Source: F1000Res. 2026 Aug 19;15:916. Originally published 2026 Jun 11. [Version 2] doi: 10.12688/f1000research.175496.2 (PMC13562934; doi:10.12688/f1000research.175496.2)
Supplement: Peer review history

# Reviewer report: Reviewer response for version 2

**DOI:** 10.5256/f1000research.208155.r512583

**Published:** 2026-09-04

**Authors:** Dr. Abdulaleem Al-Othmani

**Affiliations:** 1 School of Computer Science and Informatics, De Montfort University, Leicester, England, UK

I am happy with the revised article and the corrections made. Happy to accept/approve this submission.

Is the work clearly and accurately presented and does it cite the current literature?

Yes

If applicable, is the statistical analysis and its interpretation appropriate?

Yes

Are all the source data underlying the results available to ensure full reproducibility?

Yes

Is the study design appropriate and is the work technically sound?

Yes

Are the conclusions drawn adequately supported by the results?

Partly

Are sufficient details of methods and analysis provided to allow replication by others?

Yes

Reviewer Expertise:

Cyber Security, Mobile Communication, Signal Processing, and Machine Learning

I confirm that I have read this submission and believe that I have an appropriate level of expertise to confirm that it is of an acceptable scientific standard.

# Reviewer report: Reviewer response for version 2

**DOI:** 10.5256/f1000research.208155.r512584

**Published:** 2026-08-20

**Authors:** Assistant Prof. Ali Al-Juboori

**Affiliations:** 1 School of Theoretical and Applied Science (TAS), Ramapo College of New Jersey, Mahwah, New Jersey, USA

I reviewed the revised version of the manuscript, and the authors addressed all my concerns very well. I recommend accepting the paper as it is.

Is the work clearly and accurately presented and does it cite the current literature?

Yes

If applicable, is the statistical analysis and its interpretation appropriate?

Not applicable

Are all the source data underlying the results available to ensure full reproducibility?

Partly

Is the study design appropriate and is the work technically sound?

Yes

Are the conclusions drawn adequately supported by the results?

Yes

Are sufficient details of methods and analysis provided to allow replication by others?

Partly

Reviewer Expertise:

Data Communication, Computer Networking, Wireless Communication.

I confirm that I have read this submission and believe that I have an appropriate level of expertise to confirm that it is of an acceptable scientific standard.

# Reviewer report: Reviewer response for version 1

**DOI:** 10.5256/f1000research.193483.r493151

**Published:** 2026-07-17

**Authors:** Dr. Abdulaleem Al-Othmani

**Affiliations:** 1 School of Computer Science and Informatics, De Montfort University, Leicester, England, UK

This is a really timely and relevant experimental study. The focus on real-world 5G and FSS coexistence in Malaysia, particularly using actual indoor measurements rather than just simulations, is a definite strength. The structure is logical, the measurement-based approach is appropriate, and making the data open is a great move that adds significant value to the community. The work contributes useful empirical data for practical deployment contexts.

 That said, I do have a number of corrections and suggestions for enhancement that I believe would help strengthen the manuscript’s clarity, presentation, and completeness. My detailed corrections and suggestions for enhancement are as follows:

**Corrections:**

 1. To ensure replicability of the measurement, it is essential to add brief methodological details (Section 2: Experimental Setup, pp. 3-4) to:

    -  Clearly describe the measurement procedure, including whether measurements were repeated (number of trials per location), duration of each measurement, and sampling approach.

    -  Clarify data processing steps, e.g., whether values shown in Figures 7-12 (pp. 7-10) are instantaneous readings, averaged values, or smoothed outputs from the spectrum analyser.

 2. Revise ambiguous or unclear sentences (Abstract, p. 1; Results, pp. 6-11)

    -  The Abstract (p. 1) contains unclear or disjointed sentences (e.g., incomplete statement on “fine-tuning exclusion zones”).

    -  The Results section (pp. 6–11) includes unclear phrasing such as “in the same lap as the 5G transmission station” and “smooth mode”

    -  The explanations around RBW and VBW are repeated in the last section of p. 4. you could easily remove these repeated statements.

 3. Revise the conclusion to reflect that findings apply to the specific indoor scenario and measurement conditions tested.

**Recommended Enhancements**

 1. A general comment is that the English language and technical writing quality should be revisited throughout the manuscript. The structure of the paper is sound, however, there are recurring issues with grammar, sentence construction, and inconsistent terminology.

 2. Consider adding a short note on study limitations (end of Section 4: Conclusions, p. 13) to acknowledge single indoor environment and limited spatial/propagation diversity.

**In summary**, this manuscript provides a useful and relevant contribution to the study of 5G-FSS coexistence. The work is technically sound, and the conclusions are generally supported by the results. The issues identified are minor and primarily relate to clarity, completeness, and presentation of results.

Is the work clearly and accurately presented and does it cite the current literature?

Yes

If applicable, is the statistical analysis and its interpretation appropriate?

Yes

Are all the source data underlying the results available to ensure full reproducibility?

Yes

Is the study design appropriate and is the work technically sound?

Yes

Are the conclusions drawn adequately supported by the results?

Partly

Are sufficient details of methods and analysis provided to allow replication by others?

Yes

Reviewer Expertise:

Cyber Security, Mobile Communication, Signal Processing, and Machine Learning

I confirm that I have read this submission and believe that I have an appropriate level of expertise to confirm that it is of an acceptable scientific standard, however I have significant reservations, as outlined above.

# Response

**Published:** 2026-08-06

**Authors:** Qasim Khalaf

**Affiliations:** University of Fallujah, Al-Fallujah, Al Anbar Governorate, Iraq

Reviewer 2: Dr. Abdulaleem Al-Othmani (De Montfort University)

 We are grateful to the reviewers for their constructive and encouraging assessments and for recognizing the value of a real, measurement-based coexistence study with openly released data. Both reviewers judged the study design appropriate and the work technically sound, and neither identified an error in the measured data itself. We have accepted every comment that concerns clarity, language, presentation, framing, or a clearly editorial correction, and these are now implemented as tracked changes.

 R2 · Correction 1: Methodological detail for replicability

*Reviewer: *Add procedure detail (repetitions/trials per location, duration, sampling) and clarify whether Figures 7–12 are instantaneous, averaged, or smoothed.

**Response: partially accepted (clarification). **A single clarifying sentence has been added in §2 stating that the traces in Figures 7–12 are max-hold detector readings taken directly from the spectrum analyzer, rather than instantaneous single-sweep samples or externally averaged/smoothed values—consistent with the text already present for Figure 10. The measurement location, base station and transmit-power settings, receiver-antenna specification (Table 1), LOS/NLOS geometry, distances, and analyzer settings (RBW = VBW = 1 MHz, SWT = 100 ms) are already reported, and the complete raw and processed datasets with setup/environment metadata are openly available on Figshare (DOI: 10.6084/m9.figshare.31872805). We have documented the existing procedure rather than altering the practical campaign.

 R2 · Correction 2: Ambiguous sentences

*Reviewer: *Incomplete abstract “fine-tuning exclusion zone” statement; results phrasing “in the same lap as…” and “smooth mode”; repeated RBW/VBW text on p.4.

**Response: accepted (all three). **The abstract fragment is completed: “in the same lap as the 5G transmission station” → “at the same location as the 5G transmission station”; “in the smooth mode” → “smooth and relatively flat”; and the duplicated RBW/VBW/SWT sentences are deleted, leaving a single clear explanation. All shown as tracked changes; no measured value is affected.

 R2 · Correction 3: Conclusion scope

*Reviewer: *Revise the conclusion so findings apply to the specific indoor scenario and conditions tested.

**Response: accepted. **A caveat has been added (tracked) to §4 stating that the no-interference finding applies specifically to the single indoor RekaScape C-band scenario and the measurement conditions tested and should not be generalized without further campaigns.

 R2 · Enhancement 1: Writing quality

**Response: Accepted and applied conservatively. **Clear grammatical/typographical errors are corrected as tracked changes (e.g., "transamination"→"transmission," "puls"→"pulses," “3,456 GHz”→“3.456 GHz”). Edits were deliberately limited so that no measured value, parameter, or technical statement changes; a full professional copy-edit will follow at the proof stage.

 R2 · Enhancement 2: Study limitations

**Response — accepted. **A “Study limitations” paragraph has been added (tracked) at the end of §4: single indoor environment, limited spatial/propagation diversity, one building and base-station configuration, bounded Tx–Rx distances, and the need for further campaigns before generalization.

 R2 · “Conclusions supported: Partly."

**Response. **The added scope caveat and limitations frame the conclusion strictly within the measured evidence, so the no-interference finding is now fully supported by the presented results rather than overgeneralized.

# Reviewer report: Reviewer response for version 1

**DOI:** 10.5256/f1000research.193483.r493144

**Published:** 2026-06-29

**Authors:** Assistant Prof. Ali Al-Juboori

**Affiliations:** 1 School of Theoretical and Applied Science (TAS), Ramapo College of New Jersey, Mahwah, New Jersey, USA

The manuscript addresses the problem of interference potential between 5G indoor deployments in the 3.4–3.6 GHz C-band and existing Fixed Satellite Service (FSS) operations in Malaysia. The experimental approach, using a ZTE 5G base station inside the RekaScape building with a spectrum analyzer to measure received power at multiple distances, is sound in its basic conception, and the finding that no measurable interference was detected between the indoor 5G signal and the MEASAT FSS station at 3 km is a useful empirical contribution. The work focusing on exclusion zone optimization is directly relevant to regulators and spectrum planners.

**Major Comments**

1. The abstract and introduction promise "a 5G propagation model," yet the body of the paper presents no formal propagation model. The results consist of raw spectrum analyzer readings at discrete distances. There is no path loss equation, no comparison to standard indoor propagation models (e.g., ITU-R P.1238, 3GPP TR 38.901, WINNER+), and no curve-fitting to the measured data. The authors should either (a) explicitly derive or adopt a propagation model and fit it to their measurements, report model parameters and goodness-of-fit statistics, or (b) revise the abstract and introduction to accurately describe the study as an empirical measurement campaign rather than a propagation modeling study.
2. The core claim, that no interference exists between the 5G indoor system and FSS, is supported only by the observation that signal levels at the MEASAT station were not visibly elevated. However, the manuscript does not provide: (a) the ITU-R threshold for interference into FSS receivers (I/N or C/I criteria), (b) any calculation of the aggregate interference power that would arrive at the FSS earth station from the indoor 5G emitter at 3 km, given the building attenuation and free-space path loss, or (c) any link budget. Without these, the "no interference" conclusion lacks a quantitative basis. The reader cannot determine how far below the interference threshold the measured levels were, which is the most practically important result. A formal interference link budget should be written.
3. Table 2 recommends an exclusion zone of approximately 100 m between the 5G indoor station and FSS station, and a forbidden guard band of 50–100 MHz. These values appear without derivation, simulation, or citation. How were they determined? Are they based on measurements, a regulatory recommendation, or a prior publication? The authors must either derive these values from their data and the relevant FSS protection criteria or clearly attribute them to an existing regulatory or technical source.
4. While the experimental description is reasonably detailed, it omits several parameters that would be needed to reproduce the study. For instance, the exact model of the spectrum analyzer used (referenced as "Spectrum Analyzer - 1" and "Spectrum Analyzer - 2" in Figure 2 but not named), the calibration procedure for the measurement equipment, and the number of measurement repetitions or averaging used at each distance point. Without this information, the measurement uncertainty cannot be estimated, and the study is not fully reproducible. These details should be added to Section 2.
5. The study measures signals inside the building (LOS and NLOS) and at distances up to 40 m, but the interference scenario of primary concern is leakage from indoor 5G to an outdoor FSS earth station 3 km away. The paper does not discuss building entry loss (or exit loss) for the 3.5 GHz band, which is the critical attenuation factor in this scenario. Standard values from ITU-R P.2109 or 3GPP specifications should be cited and applied to estimate the actual out-of-building signal level, and this should inform the exclusion zone derivation.

Minor Comments

1. The abstract contains an awkward sentence structure in the passage beginning "Prior to the power signal transmission ceasing the network..." This should be rewritten for clarity. The abstract should also explicitly state the main quantitative finding (e.g., the received power range across distances, and the measured signal level relative to FSS protection thresholds).
2. The introduction covers 5G history in considerable detail (mobile network generations from 1982 onward, Cisco VNI traffic projections), which does not directly motivate the study. This background can be compressed substantially, with space redirected to a more thorough review of prior coexistence studies in the 3.5 GHz band, particularly the work of Kristiadi et al. (2024, reference 9), which addresses an almost identical problem for Indonesia and should be directly compared to the present findings.
3. Figures 7 through 12 use axis labels that are difficult to read at publication resolution. The x-axis label "GHz × 10⁹" is redundant (GHz already implies ×10⁹ Hz); this should be corrected to simply "GHz." Also, Figures 13 through 17 (propagation length plots) would benefit from explicit annotations indicating the LOS-to-NLOS transition point.
4. Reference 16 (Ellis et al., "On the maximal strength of a first-order electroweak phase transition...," J. Cosmol. Astropart. Phys.) appears to be a citation error, it concerns particle cosmology and has no connection to spectrum analysis or VBW/RBW. The authors should replace this with the appropriate technical reference for the SWT equation and VBW/RBW relationships.
5. The entry "MESAT frequency bandwidths ≥ 3.700 MHz" is almost certainly a typographic error and should read "≥ 3,700 MHz" (i.e., 3.7 GHz). Please verify and correct.
6. The abstract mentions analysis of "potential Low-Noise Block (LNB) saturation," but this analysis does not appear in the body of the paper. Either the LNB saturation analysis should be included as a distinct section, or the reference to it should be removed from the abstract.

**Recommendation:**

 Major revision is required. The study addresses a valuable and policy-relevant question, and the empirical measurements are a genuine contribution. However, the manuscript needs a formal propagation model or an accurate characterization of what was actually done, a quantitative interference link budget relative to FSS protection thresholds, a derivation of the exclusion zone recommendation, correction of sign errors in the reported power values, and resolution of the LNB saturation omission.

Is the work clearly and accurately presented and does it cite the current literature?

Yes

If applicable, is the statistical analysis and its interpretation appropriate?

Not applicable

Are all the source data underlying the results available to ensure full reproducibility?

Partly

Is the study design appropriate and is the work technically sound?

Yes

Are the conclusions drawn adequately supported by the results?

Yes

Are sufficient details of methods and analysis provided to allow replication by others?

Partly

Reviewer Expertise:

Data Communication, Computer Networking, Wireless Communication.

I confirm that I have read this submission and believe that I have an appropriate level of expertise to confirm that it is of an acceptable scientific standard, however I have significant reservations, as outlined above.

# Response

**Published:** 2026-08-06

**Authors:** Qasim Khalaf

**Affiliations:** University of Fallujah, Al-Fallujah, Al Anbar Governorate, Iraq

**Reviewer 1:** Assistant Prof. Ali Al-Juboori (Ramapo College of New Jersey)

 We are grateful to the reviewers for their constructive and encouraging assessments and for recognizing the value of a real, measurement-based coexistence study with openly released data. Both reviewers judged the study design appropriate and the work technically sound, and neither identified an error in the measured data itself. We have accepted every comment that concerns clarity, language, presentation, framing, or a clearly editorial correction, and these are now implemented as tracked changes.

 R1 · Major 1: “Propagation model”

*Reviewer: *The abstract/introduction promises a 5G propagation model, but the body presents measured readings; either derive/fit a model with goodness-of-fit or reframe it as an empirical measurement campaign.

**Response: accepted via option (b). **We have reframed the claim: the abstract now reads “an empirical, measurement-based 5G propagation assessment” rather than “a 5G propagation model,” so that the manuscript describes exactly what was performed—a measurement campaign characterizing received power versus distance under indoor LOS/NLOS conditions. We have deliberately not fitted a formal path-loss model to the data, as that would convert this empirical study into a different type of paper; such model fitting is noted as future work.

 R1 · Major 2 — Quantitative interference link budget

*Reviewer: *The “no interference” claim lacks ITU-R I/N or C/I thresholds, aggregate interference power at the FSS earth station, and a link budget.

**Response: Respectfully declined as beyond the empirical scope; the observation is retained and now scenario-bounded. **The no-interference statement is an empirical observation: at the MEASAT earth station 3 km away, no measurable elevation of the FSS signal was detected under the tested indoor 5G operation, and the 5G band (3.4–3.6 GHz) is separated from the MEASAT operating range (≥ 3.7 GHz). A full regulatory link budget requires proprietary site-specific FSS receiver parameters and deployment assumptions, which were outside this study's objective and cannot be provided without unverified assumptions. We have therefore bounded the conclusion to the measured scenario (§4) and signposted the formal I/N link budget as future work, rather than change the practical result.

 R1 · Major 3: Exclusion zone and guard band

*Reviewer: *The ~100 m separation and 50–100 MHz guard band appear without derivation, simulation, or citation.

**Response: clarified (no change to the values). **A sentence has been added after Table 2 stating explicitly that these separation and guard-band figures are practical planning considerations inferred from the measured indoor signal behavior in this specific scenario, not universal regulatory limits, and that their formal derivation against FSS protection criteria (e.g., ITU-R I/N thresholds) is future work. This addition resolves the framing concern without altering the reported values.

 R1 · Major 4 — Reproducibility (instrument detail)

*Reviewer: *The spectrum-analyzer model, calibration procedure, and number of repetitions/averaging are not given.

**Response: partially accepted (clarification). **As with Reviewer 1’s Correction 1, we have clarified in §2 that Figures 7–12 are max-hold traces read directly from the analyzer. The reported analyzer display settings (RBW = VBW = 1 MHz, SWT = 100 ms) and the openly available Figshare dataset (with setup and environmental metadata) allow the measurement scenario to be reproduced in principle. We have not added a full instrument-metrology and uncertainty-budget section, as that is a different (metrological) study scope; the measured campaign itself is unchanged.

 R1 · Major 5: Building entry/exit loss

*Reviewer: *Building entry/exit loss (ITU-R P.2109 / 3GPP) for 3.5 GHz should be cited and applied to estimate out-of-building leakage toward the FSS earth station.

**Response: Respectfully declined, as it is beyond the empirical scope. **This work is a field-measurement study: the walls and indoor environment are physically part of the measured scenario, so their attenuation is already embodied in the recorded received-power behavior. Applying a generic building-loss model would be appropriate for a simulation-based extrapolation across many building types, which is precisely the future-work extension we now signpost; it is not required to validate the empirical observation reported here.

 R1 · Minor 1: Abstract clarity and quantitative finding

**Response: accepted. **The awkward sentence beginning “Prior to the power signal transmission ceasing the network…” has been rewritten for clarity, and the abstract now states a measured finding: the received power decreased with distance to approximately −74.02 dBm at 40 m, with no measurable elevation of the FSS signal at MEASAT Station 3 km away.

 R1 · Minor 2: Introduction focus

**Response: Partially accepted. **A direct comparison to Kristiadi et al. (2024, ref. 9) has been added to the Introduction, noting that the present study complements their Indonesian protection-zone analysis by contributing direct Malaysian indoor measurements. We have retained the historical context for readers new to the band; further trimming can be applied at the proof stage in agreement with the editor.

 R1 · Minor 3: Figure axis labels and LOS/NLOS annotation

**Response: accepted for the figure files. **Figures 13–17 have been regenerated as propagation-length plots in which the direct (LOS) and diffraction (NLOS) paths are explicitly marked, directly implementing the requested LOS/NLOS annotation; the redundant “GHz × 10⁹” label on Figures 7–12 will be corrected to “GHz” in the revised figure set. These are figure-image corrections and do not change any reported value or interpretation.

 R1 · Minor 4: Reference 16

**Response: accepted. **The unrelated cosmology citation has been replaced (tracked) with an appropriate spectrum-analysis reference (Keysight Technologies, “Spectrum Analysis Basics,” Application Note 150) supporting the SWT/VBW/RBW discussion.

 R1 · Minor 5: MEASAT frequency notation

**Response: accepted. **Corrected (tracked) in Table 2 to “MEASAT frequency bandwidths ≥ 3,700 MHz (3.7 GHz).” This is a typographical/units correction; the physical value (3.7 GHz), already stated in the Conclusions, is unchanged.

 R1 · Minor 6: LNB saturation

**Response: accepted. **The abstract has been reworded so that LNB saturation is presented as part of the coexistence-risk context discussed in the paper, rather than implying a dedicated LNB saturation experiment. This makes the abstract consistent with the body; a dedicated LNB compression study is noted as future work.

 R1 · Recommendation: alleged sign errors in power values

**Response: respectfully declined; values verified. **We re-checked the reported received-power values against the publicly available Figshare dataset. The negative dBm figures are correct as measured; received power at the spectrum analyzer input is naturally expressed in negative dBm, and the per-figure peak values are consistent with distance (for example, −13.41 dBm at 10 m in Figure 8 decreasing to −74.02 dBm at 40 m behind a cement wall in Figure 12). Prompted by this comment, we also reviewed the overall received-power range summarized in the Conclusions and have ensured it is reported consistently with the values shown in the figures. No change to the underlying measured data is warranted.
